# Distribution differences in prognostic copy number alteration profiles in *IDH*-wild-type glioblastoma cause survival discrepancies across cohorts

**DOI:** 10.1186/s40478-019-0749-8

**Published:** 2019-06-18

**Authors:** Toru Umehara, Hideyuki Arita, Ema Yoshioka, Tomoko Shofuda, Daisuke Kanematsu, Manabu Kinoshita, Yoshinori Kodama, Masayuki Mano, Naoki Kagawa, Yasunori Fujimoto, Yoshiko Okita, Masahiro Nonaka, Kosuke Nakajo, Takehiro Uda, Naohiro Tsuyuguchi, Junya Fukai, Koji Fujita, Daisuke Sakamoto, Kanji Mori, Haruhiko Kishima, Yonehiro Kanemura

**Affiliations:** 1 Department of Neurosurgery, Osaka University Graduate School of Medicine, 2-2 Yamadaoka, Suita, Osaka 565-0871 Japan; 2 Kansai Molecular Diagnosis Network for CNS Tumors, Osaka-City, Osaka Japan; 3 Department of Biomedical Research and Innovation Research, Institute for Clinical Research, Osaka National Hospital, National Hospital Organization, 2-1-14 Hoenzaka, Chuo-ku, Osaka-City, Osaka 540-0006 Japan; 4 Department of Neurosurgery, Osaka International Cancer Institute, 3-1-69, Otemae, Chuo-ku, Osaka-City, Osaka 541-8567 Japan; 5 Department of Pathology and Applied Neurobiology, Kyoto Prefectural University of Medicine, Kamigyo-ku Kajii-cho, Kawaramachi-Hirokoji, Kyoto-City, Kyoto 602-8566 Japan; 6 Department of Central Laboratory and Surgical Pathology, Osaka National Hospital, National Hospital Organization, 2-1-14 Hoenzaka, Chuo-ku, Osaka-City, Osaka 540-0006 Japan; 7 Department of Neurosurgery, Osaka National Hospital, National Hospital Organization, 2-1-14 Hoenzaka, Chuo-ku, Osaka-City, Osaka 540-0006 Japan; 8 Department of Neurosurgery, Kansai Medical University, 3-1 Shinmachi 2 Chome, Hirakata City, Osaka 573-1191 Japan; 9 Department of Neurosurgery, Osaka City University Graduate School of Medicine, 1-5-7, Asahi-machi, Abeno-ku, Osaka-City, Osaka 545-8586 Japan; 10 Department of Neurosurgery, Kindai University Faculty of Medicine, 337-2, Ono-higashi, Osaka Sayama-City, Osaka 589-8511 Japan; 11 Department of Neurological Surgery, Wakayama Medical University School of Medicine, Kimiidera 811-1, Wakayama, 641-0012 Japan; 12 Department of Neurosurgery, Hyogo College of Medicine, 1-1 Mukogawa, Nishinomiya, Hyogo 663-8501 Japan; 13 Department of Neurosurgery, Kansai Rosai Hospital, 3-1-69, Inabasou, Amagasaki City, Hyogo 660-5811 Japan

**Keywords:** Glioblastoma, Copy number alteration, *EGFR*, *CDKN2A*, *PTEN*

## Abstract

The diagnosis and prognostication of glioblastoma (GBM) remain to be solely dependent on histopathological findings and few molecular markers, despite the clinical heterogeneity in this entity. To address this issue, we investigated the prognostic impact of copy number alterations (CNAs) using two population-based *IDH*-wild-type GBM cohorts: an original Japanese cohort and a dataset from The Cancer Genome Atlas (TCGA). The molecular disproportions between these cohorts were dissected in light of cohort differences in GBM. The Japanese cohort was collected from cases registered in Kansai Molecular Diagnosis Network for CNS tumors (KNBTG). The somatic landscape around CNAs was analyzed for 212 KNBTG cases and 359 TCGA cases. Next, the clinical impacts of CNA profiles were investigated for 140 KNBTG cases and 152 TCGA cases treated by standard adjuvant therapy using temozolomide-based chemoradiation. The comparative profiling indicated unequal distribution of specific CNAs such as *EGFR*, *CDKN2A*, and *PTEN* among the two cohorts. Especially, the triple overlap CNAs in these loci (triple CNA) were much higher in frequency in TCGA (70.5%) than KNBTG (24.3%), and its prognostic impact was independently validated in both cohorts. The KNBTG cohort significantly showed better prognosis than the TCGA cohort (median overall survival 19.3 vs 15.6 months). This survival difference between the two cohorts completely resolved after subclassifying all cases according to the triple CNA status. The prognostic significance of triple CNA was identified in *IDH*-wild-type GBM. Distribution difference in prognostic CNA profiles potentially could cause survival differences across cohorts in clinical studies.

**Electronic supplementary material:**

The online version of this article (10.1186/s40478-019-0749-8) contains supplementary material, which is available to authorized users.

## Introduction

The recent comprehensive molecular analysis of glioblastoma (GBM), including The Cancer Genome Atlas (TCGA) projects, has revealed the tumor genetic landscape and various functional relations between genes and pathways in tumorigenesis [4]. Several molecular alterations by somatic mutation, genomic rearrangement, and copy number alteration (CNA) have been shown to be closely involved in GBM. One of the major interests with regards to these genetic changes is towards the availability of prognostic and/or diagnostic stratification or potential targetability of such genetic changes in the development of novel therapeutic agents.

The most important molecular change in glioma is *IDH1/2* mutations [24, 29], which define biological characteristics in glioma through the change of global DNA methylation and histone modification. These mutations have been well validated to be favorable prognostic factor in GBM patients in several studies [22]. In the 2016 WHO Classification of Tumors of the Central Nervous System (CNS WHO), *IDH*-wild-type GBM came to be regarded as a more delineated entity: the most common and malignant astrocytic glioma [19]. O^6^-methylguanine-DNA-methyltransferase (*MGMT*) methylation status is also regarded as a prognostic/predictive marker [13, 26], while it has little value as a diagnostic marker and does not define any subtypes of GBMs with distinct molecular features [17].

The CNA in GBM is another significant somatic alteration which often shows a distinctive landscape with synchronous genomic gains and/or losses. Long before discovering *IDH1/2* mutation, a chromosomal aberration in GBM was initially detected by gain of *EGFR* [18], which was subsequently followed by various CNAs. CNAs such as gain in chromosome 7 and loss of chromosomes 9 and 10 were regarded as hallmarks especially for *IDH*-wild-type GBM [10], and have become increasingly recognized to play a key role in GBM oncogenic pathways, according to CNA profiles by transcriptome subtypes [27]. Some CNAs, such as loss of chromosome 10 [23] and *NFKBIA* deletion [2], were also reported to be solely associated with survival in GBM patients. Nevertheless, since their correlation with survival has not been fully validated in the subsequent studies, no consensus has been reached as to which CNA has a universal prognostic value beyond the WHO grading of GBM.

It remains a problem that the diagnosis and prognostication of GBM largely depend on histopathological findings and few molecular features in *IDH1/2* and *MGMT* [28], despite the heterogenous clinical courses of *IDH*-wild-type GBM and even in the subsets with the same status of *MGMT* methylation. For achieving accurate prognostic stratification in GBM, we investigated molecular distribution of CNAs and its relationship to clinical outcome using two independent population-based cohorts of GBM including an original Japanese cohort and a large dataset from TCGA. When targeting two different population from east Asia and TCGA, another concern is the geographical diversity in clinical and molecular profiles of GBMs. For instance, the lower *EGFR* amplification rates of GBM patients from Asia were recently reported during a screening for two randomized GBM trials with depatux-m: INTELLANCE1 and INTELLANCE2 (10.1093/annonc/mdx366.002). Therefore, a comparative study between two cohorts should deserve special consideration to help in identifying the cohort disproportion in GBM practice.

Here, we examined the survival impact of CNAs in Japanese and TCGA GBM cohorts. The differences of molecular profiles and clinical outcome were carefully investigated between these two cohorts. This investigation conclusively showed that a combination of *EGFR*, *CDKN2A,* and *PTEN* CNA status had a prognostic impact in GBM patients, and that the differences in the frequencies of these molecular profiles resulted in the different survival between the two cohorts.

## Materials and methods

### Study design

**Fig. 1:**
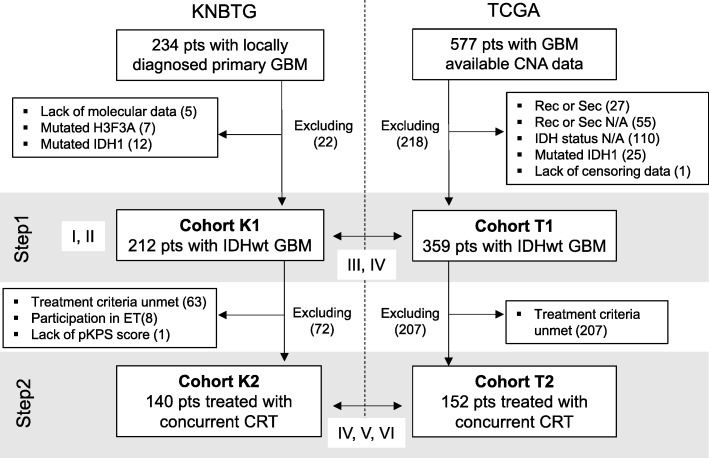
Study design and patient selection. This study consisted of two steps (Step1 and 2). In Step1, 212 primary GBM cases in KNBTG enrolled as Cohort K1. From TCGA dataset, 359 cases conclusively diagnosed with primary IDH-wild-type GBM were selected as Cohort T1. In Step2, 140 patients from Cohort K1 and 152 patients from Cohort T1 were further extracted as Cohort K2 and T2, who were concurrently treated with TMZ and RT. Targeting for each cohort or step, the analyses were conducted in the Roman numerical order as follows: I, somatic genetic landscape of GBM; II, interaction among each genetic alteration; III, comparison of frequency of genetic alterations among cohorts; IV, survival difference among cohorts; V, exploration of prognostic biomarkers; VI, survival analysis between cohorts by adjustment with common prognostic biomarkers. Abbreviations: CRT, chemoradiation therapy; ET, experimental treatment; N/A, not available; pKPS, preoperative KPS; pts., patients; Rec or Sec, recurrent or secondary GBM; wt, wild type

The analyses of this retrospective study included two steps. The detail of this study design and flowchart of patient selection are provided below and are summarized in Fig. 1. Two cohorts were collected as described below: the Japanese and TCGA cohorts. Briefly, Step1 aimed to investigate the somatic landscape including CNAs in the primary *IDH* wild-type GBM and compared the somatic landscapes of the two cohorts with each other. In Step2, we investigated the clinical impact of CNA profiles in primary *IDH* wild-type GBM cases treated with chemoradiotherapy with TMZ after initial surgery using the Japanese and TCGA cohorts [26]. The Japanese cohorts collected for Step1 and 2 analyses were Cohort K1 and K2, respectively. Similarly, TCGA cohorts subjected to each step were Cohort T1 and T2, respectively.

### A Japanese GBM cohort of Kansai molecular diagnosis network for CNS tumors (KNBTG)

Japanese cohort was collected from the cases registered in Kansai Molecular Diagnosis Network for CNS tumors (KNBTG): a consortium where neuro-oncologists, neurosurgeons, pediatricians, pathologists, and basic scientists are conducting cooperative researches for malignant brain tumor [25]. The researchers are affiliated to university hospitals, regional medical centers, and research institutes mainly in the Kansai area of Western Japan. This network routinely collects glioma cases registered from the participating institutions. Tumor samples and clinical information of patients treated at the affiliated institutions are collected and registered in this data bank after obtaining written informed consent. The detailed clinical information including preoperative Karnofsky Performance status (KPS), extent of resection, and adjuvant therapy are routinely obtained from each institution. Since this is a population-based study in Japan, subjects are exclusively composed of East Asians and under the universal health care insurance system of Japan.

The initial screening criteria from enrolled gliomas in KNBTG was as follows: local diagnosis of primary GBM and availability of genomic DNA for molecular analysis of the naïve specimens. Primary GBM was clinically regarded as the GBM arising de novo, with no known lower-grade precursor lesion. The inclusion criteria for Cohort K1 was as follows: central review of a histopathological diagnosis of *IDH*-wild-type GBM based on the 2016 CNS WHO, molecular data available for analysis, and absence of *IDH1/2* and *H3F3A* mutation, and 1p/19q codeletion. Out of 234 primary GBM initially collected from seven institutions participating in this study, 212 cases were enrolled as Cohort K1.

To analyze the prognostic impact of CNAs, cases in which patients underwent radiotherapy (RT) of 50–65 Gy and concurrent TMZ were further selected from Cohort K1. Those treated with either RT alone, TMZ alone, or short-course RT plus TMZ were excluded from any survival analysis. Cases ineligible for treatment criteria and lacking in KPS score were excluded. In addition, those participating in clinical studies for testing experimental treatment add-on to RT plus TMZ *(n* = *8)* were excluded. Finally, 140 cases were available for Step2 analysis as Cohort K2.

### Central pathology review of KNBTG cohort

All molecular-pathological diagnosis of *IDH*-wild-type GBM eligible for this investigation had reached an agreement with an experienced neuropathologist (Y.K.) and was according to the 2016 WHO classification for central nervous system tumors.

### Molecular analysis of KNBTG cohort

All tissue specimens were obtained at the time of the initial treatment before chemoradiation between December 2006 and November 2017. Tumor genomic DNA was extracted with NucleoSpin Tissue kit (Macherey-Nagel, Inc., Bethlehem, PA) or DNeasy Blood & Tissue Kit (Qiagen, Tokyo, Japan), according to the manufacturer’s protocol. The same batch of DNA samples was used for each technique as follows.

Mutational status of *IDH1/2*, *TERT* promoter, *H3F3A*, *HIST1H3B*, and *TP53* were determined using the Sanger technique. The details of Sanger sequencing have been previously reported [1] and is additionally provided in the supplementary information (SI). The methylation status of the *MGMT* promoter was assessed using quantitative methylation specific PCR (qMSP) following the bisulfite modification of tumor genomic DNA. The details of the qMSP protocol for testing *MGMT* methylation are described in SI.

To assess CNAs in GBM, we performed Multiplex Ligation-dependent Probe Amplification (MLPA) using the SALSA MLPA KIT P105 (version D2) and P088 (version C2), in accordance with the manufacturer’s protocol (MRC Holland, Amsterdam, Netherland) [15]. The P105 kit is designed to detect CNAs typical in gliomas, and includes probes against *PDGFRA*, *EGFR*, *CDKN2A*, *PTEN*, *TP53*, *CDK4*, *MDM2*, and *NFKBIA* genes. P088 kit was designed to assess mainly 1p/19q codeletion, and we used this kit only for the *IDH*-mutated-GBM to exclude oligodendroglial tumors. Based on previous publications, the CNA category was classified by the following thresholds: homozygous deletion (x ≤ 0.4), hemizygous deletion (0.4 < x ≤ 0.7), gain (1.3 ≤ x < 2.0), amplification (x ≥ 2.0) [16]. Any other details of the MLPA protocol and its data analysis to assess CNAs are described in SI.

### The cancer genome atlas (TCGA)

An independent and extensive molecular data and clinical information including survival data and treatment of GBM in TCGA (*n* = 577) were collected from cBioPortal for Cancer Genomics (https://cbioportal.org) [6, 11] and the supplemental data of the previous publication by TCGA [3, 5]. Based on the racial distribution available from cBioPortal (*n* = 550), whites accounted for a much higher percentage (88.5%, *n* = 487) than blacks (9.1%, *n* = 50) and Asians (2.4%, *n* = 13).

Out of 577 cases initially collected, 359 cases were enrolled as Cohort T1, who were conclusively diagnosed with primary *IDH*-wild-type GBM. Recurrent, secondary, or *IDH*-mutant-GBMs along with those that lacked any such information were differentially excluded from this step. For the purpose of survival analysis in Step1, only one case was additionally excluded due to a lack of censoring data.

From Cohort T1, 152 cases receiving TMZ chemoradiation as initial treatment were selected for external validation (Cohort T2) [3]. Those initially treated with either RT alone, TMZ alone, or alkylating chemotherapy other than TMZ, along with those where any such information was unavailable, were excluded. Thus, the inclusion criteria of the cohort from TCGA were similar to that for the Japanese cases in each step.

### Molecular and clinical data acquisition from a larger dataset in TCGA

All data regarding CNA in each patient from TCGA were downloaded from the online resource provided in cBioPortal for Cancer Genomics on July 15th, 2018. To identify CNA profiles, we employed putative copy number calls generated by the GISTIC or RAE algorithms in the portal, such as “-2”, “-1”, “0”, “1”, and “2” [6, 11]. These acquired calls were converted to foregoing categories as follows: − 2 to homozygous deletion, − 1 to hemizygous deletion, 0 to neutral, 1 to gain, and 2 to amplification. The mutation status in *TP53* was also downloaded from the portal. Further molecular data about *MGMT* methylation and various clinical information were available from the supplemental data of the previous publication by TCGA [3, 5]. *IDH* mutational status could be obtained from both data sources and obtainable results were fully cross-validated with each other. There were few data regarding *TERT* promoter mutation in any of the data sources.

### Statistical analysis

All statistical analyses were performed using a JMP Pro version 13 software (SAS Institute, Cary, NC, USA). The difference was considered significant if the *p*-value was < 0.05.

Patients were subdivided into two groups on the basis of age (≥ 65 or < 65 years), preoperative KPS (≤ 70 or 80–100%), and extent of resection (< 90% or 90–100%) for the purposes of statistical analysis.

Pair associations of molecular variables were evaluated using a two-tailed Fisher’s exact test and computing the log odds ratio (LOR). Concurrent with significant correlation (*p* <  0.05, Fisher’s exact), LOR below − 2.0 and above 2.0 were respectively defined as association toward mutual exclusivity and co-occurrence. LOR from − 2.0 to − 1.5 and 1.5 to 2.0 were defined to have a tendency for these associations.

For survival analysis, overall survival (OS) was defined as the interval between the initial operative day and the date of either death or the last follow-up date on which the patient was known to be alive, with a cut-off date of 28 February 2018 for the KNBTG cohort. Patients who were still alive at the last follow-up were considered as a censored event. The survival data were analyzed using the log-rank test and Cox regression analyses. In Cox analysis, the hazard ratio (HR) and p-value were computed. Variables with a significant p-value in univariate analysis were subsequently used to build a multivariate Cox model. Stepwise procedure was used for constructing multivariate Cox regression model for survival. After excluding non-significant factor one by one in the multivariate analysis, the remaining variables (p <  0.05) were considered to be independent predictors of survival. Extent of resection and *TERT* promoter mutation status were not considered in Cox regression model for the TCGA cohort because of a lack of information for all or the majority of patients in this dataset.

## Results

### Genetic characteristics of cohort K1 (step 1)

*TERT* promoter mutation, *MGMT* promoter methylation, and *TP53* mutation were observed in 125 cases (59.0%), 98 cases (46.2%), and 80 cases (37.7%), respectively. The major CNA, frequently observed in Cohort K1, were *CDKN2A* deletion (62.3%), *EGFR* amp/gain (56.1%), and *PTEN* deletion (44.3%), as shown in Table 1. Association or tendency toward co-occurrence was observed among *TERT* promoter mutation, *EGFR* gain/amp, and *PTEN* deletion with each other. *TERT* promoter mutation also had a tendency of mutual exclusivity with *PDGFRA* amplification (LOR − 1.97, p<0.001). Association toward mutual exclusivity was observed in the pairs of *PDGFRA* amplification/ *EGFR* amplification, *CDKN2A* deletion/*CDK4* amplification, and *TP53* mutation/*MDM2* amp/gain. *MGMT* methylation status was not significantly associated with any genetic alterations. These mutually exclusive or co-occurring sets are shown in Additional file 1: Figure S1. In the case of pair association, the details for computed LORs and *p*-values from the Fisher’s exact test are provided in Additional file 2: Table S3 and S4.

**Table 1:** Comparison of molecular and clinical characteristics of patients with *IDH*-wild-type GBM

			Step 1		Step 2	
			Cohort K1 (*n* = 212)	Cohort T1 (*n* = 359)	Cohort K2 (n = 140)	Cohort T2 (n = 152)
Clinical status	Age (years) at diagnosis	Median (range)	67.0 (18–93)	61.0 (18–89)	64.0 (18–82)	59.0 (18–86)
		elderly (≥65)	125 (59.0%)	138 (38.4%)	67 (47.9%)	41 (27.0%)
	Gender	Male	114 (53.8%)	221 (61.6%)	77 (55.0%)	93 (61.2%)
		Female	98 (46.2%)	138 (38.4%)	63 (45.0%)	59 (38.8%)
	Preoperative KPS (%)	80–100	99 (47.4%)	195 (71.2%)	79 (56.4%)	100 (79.4%)
		0–70	110 (52.6%)	79 (28.8%)	61 (43.6%)	26 (20.6%)
		N/A	3	85	0	26
	Extent of resection	≥ 90%	104 (49.8%)	–	68 (48.6%)	–
		N/A	3	–	0	–
*TERT* promoter		Mut	125 (59.0%)	–	81 (57.9%)	–
		N/A	0	–	0	–
*MGMT* promoter		Met	98 (46.2%)	110 (41.5%)	59 (42.1%)	56 (44.8%)
		N/A	0	94	0	27
*TP53*		Mut	80 (37.7%)	60 (26.7%)	53 (37.9%)	22 (24.4%)
		N/A	0	134	0	62
CNA	*PDGFRA*	Amp	33 (15.6%)	53 (14.8%)	20 (14.3%)	19 (12.5%)
		Gain	10 (4.7%)	20 (5.6%)	8 (5.7%)	11 (7.2%)
	*EGFR*	Amp	54 (25.5%)	181 (50.4%)	39 (27.9%)	76 (50.0%)
		Gain	65 (30.7%)	159 (44.3%)	39 (27.9%)	67 (44.1%)
	*CDKN2A*	Homo	79 (37.3%)	210 (58.5%)	58 (41.4%)	87 (57.2%)
		Hemi	53 (25.0%)	61(17.0%)	30 (21.4%)	23 (15.1%)
	*PTEN*	Homo	4 (1.9%)	41 (11.4%)	3 (2.1%)	14 (9.2%)
		Hemi	90 (42.5%)	300 (83.6%)	58 (41.4%)	132 (86.8%)
	*CDK4*	Amp	23 (10.8%)	57 (15.9%)	13 (9.3%)	19 (12.5%)
		Gain	7 (3.3%)	33 (9.2%)	7 (5.0%)	17 (11.2%)
	*MDM2*	Amp	18 (8.5%)	33 (9.2%)	10 (7.1%)	12 (7.9%)
		Gain	3 (1.4%)	28 (7.8%)	3 (2.1%)	15 (9.9%)
	*NFKBIA*	Homo	1 (0.5%)	0 (0.0%)	0 (0.0%)	0 (0.0%)
		Hemi	46 (21.7%)	109 (30.4%)	33 (23.6%)	45 (29.6%)
	*TP53*	Homo	8 (3.8%)	6 (1.7%)	5 (3.6%)	3 (2.0%)
		Hemi	78 (36.8%)	56 (15.6%)	50 (35.7%)	22 (14.5%)
		Mut/Del	122 (57.5%)	96 (38.6%)	80 (57.1%)	39 (38.6%)
	Triple CNA	Triple	51 (24.1%)	253 (70.5%)	34 (24.3%)	103 (67.8%)
		Non triple	161 (75.9%)	106 (29.5%)	106 (75.7%)	49 (32.2%)

*Abbreviations*: *Amp* Amplification, *Del* homozygous and/or hemizygous deletion, *Hemi* hemizygous deletion, *Homo* homozygous deletion, *Met* methylated, *Mut* mutated, *Mut/Del* Mut or Del, *N/A* not available

### Frequencies of several genetic abnormalities differed across the cohorts KNBTG and TCGA (Step 1)

The molecular characteristics of each cohort are shown in Table 1. The details for each case from Cohort K1 and T1 are provided in Additional file 2: Table S1 and S2, respectively.

**Fig. 2:**
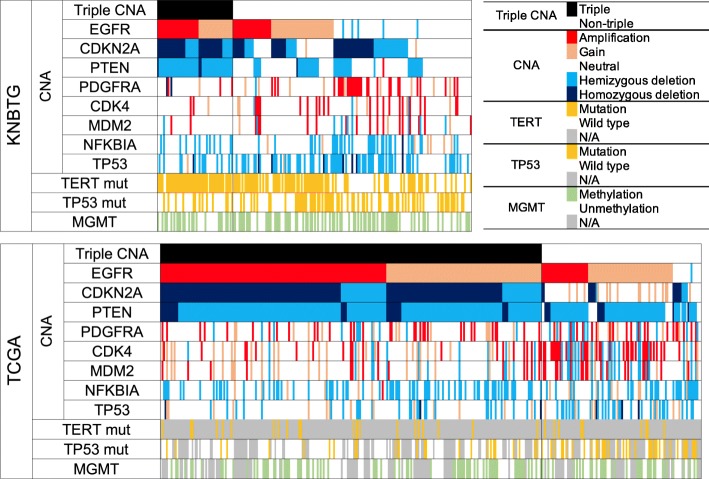
Genetic distribution in IDH-wild-type GBM among the two cohorts. The diagram shows the landscape of the molecular characteristics of IDH-wild-type GBM from KNBTG (upper figure) and TCGA (lower figure), which are sorted by CNAs in *EGFR*, *CDKN2A*, and *PTEN*. The triple overlap CNAs in *EGFR*, *PTEN*, and *CDKN2A* (triple CNA) proved to be approximately three-fold higher in frequency in TCGA. Abbreviations: Alteration, mutation and/or copy number alteration; mut, mutation; N/A, not available

The frequencies of genetic abnormalities, especially in *EGFR*, *CDKN2A*, and *PTEN,* in Cohort K1 were lower than that in Cohort T1, with the difference in excess of 20%. The triple overlapping presence of CNAs in *EGFR*, *CDKN2A*, and *PTEN* (termed triple CNA) especially showed the apparent distinction between Cohort K1 (24.1%) and T1 (70.5%). The frequencies of *TP53* mutation and deletion were higher in Cohort K1 (57.5%) than in T1 (38.6%). The distribution of *MGMT* methylation status between the two cohorts was roughly equivalent (46.2% versus 41.5%). The patient molecular status of each cohort is shown in Fig. 2.

### Comparative survival analysis revealed favorable outcome in the Japanese cohort (K1) (Step1)

Cohort K1 showed significantly longer survival (median OS, 16.7 months) than Cohort T1(median OS, 12.9 months) (*p* <  0.001, log-rank test) (Additional file 1: Figure S2). We compared the clinical characteristics of K1 and T1 to identify the factors that caused the survival difference. As shown in Table 1, the proportion of elderly patients (≥ 65) in Cohort K1 (59.0%) was higher than that in T1 (38.4%). Although KPS status in TCGA was not fully obtained, patients with low preoperative KPS (≤ 70%) were more common in Cohort K1 (52.6%) versus T1 (28.8%). Unfavorable prognostic factors were more frequent in the Japanese cohort, contrary to our first hypothesis that the Japanese cohort harbor clinical advantages for survival. Therefore, we formulated another hypothesis that the differences in genetic backgrounds including CNAs may cause the discrepancies of clinical outcomes.

### Exploration of potential prognostic biomarkers based on CNAs (Step 2)

**Fig. 3:**
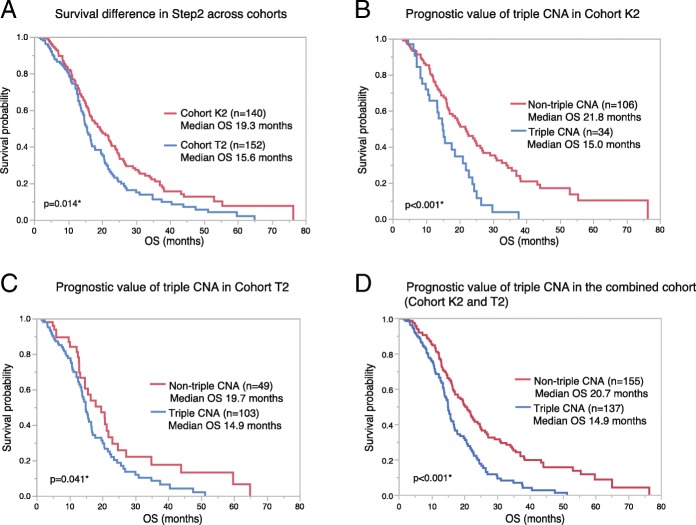
Survival difference in Step2 across cohorts and prognostic value of triple CNA. **a**. Kaplan–Meier estimates of OS in Step2 for Cohort K2 (*n* = 140) and T2(*n* = 152) are shown. OS of Cohort T2 showed significantly shorter survival than that of Cohort K2 (*p* = 0.014) as well as in Step1, even after the adjustment of postoperative treatment background. b-c. Kaplan–Meier estimates of OS according to triple CNA are shown on each cohort: Cohort K2 (n = 140) (**b**) and Cohort T2 (n = 152) (**c**). Cases with triple CNA significantly showed a worse prognosis than that without triple CNA both in Cohort K2 (*p* < 0.001, Log-rank test) and Cohort T2 (*p* = 0.041). Median OS of cases with triple CNA were roughly 15 months that was comparable in the two cohorts. **d**. In the combined cohort of 292 patients (both Cohort K2 and T2), triple CNA was also a negative prognostic indicator (p < 0.001, log-rank test). Abbreviations: OS, overall survival; triple CNA, the triple overlap of copy number alterations in *EGFR*, *PTEN* and *CDKN2A*

To evaluate the impact of molecular profiles on the survival difference across the cohorts, we collected the cases with homogenous treatment background from KNBTG and TCGA (Cohort K2 and T2, respectively). Similar to that in Step1, OS of Cohort T2 (median OS, 15.6 months) was significantly shorter than that of Cohort K2 (median OS, 19.3 months) (*p* = 0.014) (Fig. 3a). Thus, population from KNBTG showed longer survival than that from TCGA, regardless of postoperative TMZ chemoradiation. When conducting comparative profiling of these cohorts (Table 1), patients with advanced age and lower KPS were similarly more frequent in Cohort K2. We concluded that these cohorts (K2 and T2) were suitable for investigating the impact of molecular profiles on the survival difference.

The prognostic impact of each clinical or molecular factors were examined in Cohort K2 (Table 2, Additional file 2: Table S5). In univariate Cox regression analysis, advanced age (≥ 65) at diagnosis, preoperative KPS ≤ 70%, extent of resection < 90%, *MGMT* unmethylation, and *TERT* promoter mutation were associated with unfavorable prognosis. Focusing on CNAs, *PTEN* deletion and *NFKBIA* deletion were associated with shorter survival, while *EGFR* amp/gain, *CDKN2A* deletion, and *TP53* deletion were not associated with OS. Note that the cases with triple CNA significantly showed a worse prognosis (HR 2.134, *p* = 0.002). Median OS was significantly different between cases in Cohort K2 with triple CNA (15.0 months) and without triple CNA (21.8 months) (*p* <  0.001, log-rank test). Kaplan–Meier estimates according to triple CNA is shown in Fig. 3b. Next, we proceeded to multivariate analysis using a model including the possible explanatory variables in univariate analysis (age at diagnosis, preoperative KPS, extent of resection, *MGMT*, and probable CNA profiles). After excluding non-significant factors by a stepwise procedure, triple CNA was found to be an independent prognostic factor along with age, extent of resection, *NFKBIA*, and *MGMT* in Cohort K2 (Table 2).

**Table 2:** Cox proportional hazards models in Step 2

		Univariate			Multivariate		
		HR	95% CI for HR	p-value	HR	95% CI for HR	*p*-value
Cohort K2 (*n* = 140)							
Age^a^		1.027	1.009–1.047	0.002^†^	1.035	1.016–1.056	< 0.001^†^
KPS	≤70%	1.611	1.082–2.391	0.019^†^	excluded by factor selection with step-wise method		
	≥80%	Ref	–	–			
Extent of resection	< 90%	1.555	1.051–2.310	0.027^†^	1.860	1.246–2.790	0.002^†^
	≥90%	Ref	–	–	Ref		
* MGMT* promoter	Un-Met	2.037	1.351–3.123	< 0.001^†^	2.447	1.601–3.808	< 0.001^†^
	Met	Ref	–	–	Ref		
* PTEN*	Del	1.552	1.041–2.314	0.031^†^	excluded by factor selection with step-wise method		
	Neutral	Ref	–	–			
* NFKBIA*	Del	1.613	1.016–2.490	0.043^†^	1.886	1.181–2.930	0.009^†^
	Neutral	Ref	–	–	Ref		
Triple CNA	Triple	2.134	1.348–3.303	0.002^†^	2.361	1.475–3.702	< 0.001^†^
	Non-triple	Ref	–	–	Ref		
Cohort T2 (*n* = 152)							
Age^a^		1.023	1.005–1.041	0.009^†^	excluded by factor selection with step-wise method		
* MGMT*	Un-Met	2.316	1.419–3.855	< 0.001^†^	2.320	1.422–3.860	< 0.001^†^
	Met	Ref			Ref		
* NFKBIA*	Del	1.767	1.118–2.735	0.016^†^	excluded by factor selection with step-wise method		
	Neutral	Ref					
Triple CNA	Triple	1.576	1.027–2.489	0.037^†^	1.736	1.053–2.980	0.030^†^
	Non-triple	Ref			Ref		

*Abbreviations*: *CI* confidence interval, *Del* homozygous and/or hemizygous deletion, *Met* methylated, *Ref* Reference: ^a^HR is for each 1 year increase. ^†^Statistically significant (*p* < 0.05)

In univariate Cox regression analysis for Cohort T2, age ≥ 65 at diagnosis, *MGMT* unmethylation, *NFKBIA* deletion, and triple CNA were unfavorable prognostic factors, similar to the results of the analysis using Cohort K2 (Table 2, Additional file 2: Table S5). Median OS varied widely between cases in Cohort T2 with triple CNA (14.9 months) and without triple CNA (19.7 months) (*p* = 0.041, log-rank test). Kaplan–Meier estimates according to triple CNA is shown in Fig. 3c. In a multivariate Cox model incorporating the significant factors in univariate analysis (age, *MGMT*, *NFKBIA*, and triple CNA), *MGMT* methylation and triple CNA remained as independent prognostic factors (Table 2). Thus, the clinical significance of *MGMT* methylation and triple CNA was validated in both Cohorts K2 and T2. The details regarding univariate analysis for each molecular variable is shown in Additional file 2: Table S5. In the combined cohort of 292 patients (both Cohort K2 and T2), triple CNA was also the negative prognostic indicator (*p* < 0.001, log-rank test) (Fig. 3d).

### Adjustment for common prognostic biomarkers in survival analysis

**Fig. 4:**
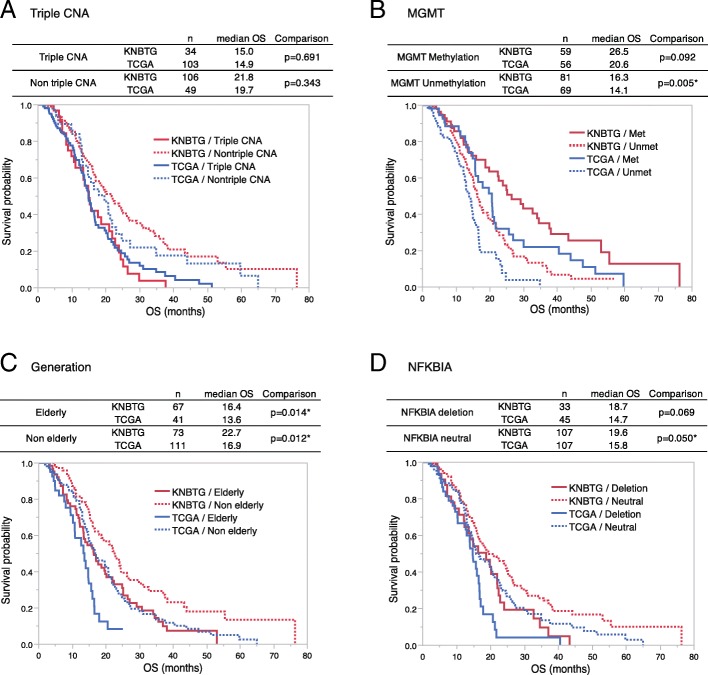
Kaplan-Meier analysis of overall survival between KNBTG and TCGA after stratification by the common prognostic biomarkers. Population in Step2 (both Cohort K2 and T2) were subdivided into two subgroups either present or absent of triple CNA, *MGMT* methylation, elderly, or *NFKBIA* deletion, respectively. OS comparison between KNBTG and TCGA for each subgroup was performed with log-rank test and Kaplan–Meier plot are shown. The status of biomarkers is recognizable by solid or dotted line. Red curves indicate KNBTG, while blue ones indicate TCGA. **a**. The solid curves (triple CNA) are almost overlapped. There is also crossover between dotted curves (non-triple CNA). The statistical discrepancies of OS between KNBTG and TCGA resolved completely both in triple CNA subgroup (*p* = 0.691, Log-rank test) and non-triple CNA subgroup (*p* = 0.343) as shown in a table above. **b**-**d**. Even after being stratified by other prognostic factors (*MGMT*, generation, and *NFKBIA*), survival differences remained significant in at least one of the subgroups (either present or absent of these factors) as shown in each table above. Abbreviations: KNBTG, Kansai Molecular Diagnosis Network for CNS tumors; Met, methylated; OS, overall survival; TCGA, The Cancer Genome Atlas; triple CNA, the triple overlap of copy number alterations in *EGFR*, *PTEN* and *CDKN2A*; Unmet, unmethylated

Here, all cases in Step2 (*n* = 292) were subdivided into subgroups with or without the common prognostic markers in univariate analysis (Age, *MGMT*, *NFKBIA*, and triple CNA) to apply their adjustment, and Kaplan-Meier curve comparison for cohorts with log-rank test was shown in Fig. 4 (four curves for each biomarker). Remarkably, the statistical discrepancies of OS between KNBTG and TCGA completely resolved after adjusting by the triple CNA status (Fig. 4a). However, even after adjustment by other prognostic factors including *MGMT* methylation status, generation, and *NFKBIA* status, survival differences remained significant in at least one of the subgroups representing presence or absence of these factors. (Fig. 4b-d) Accordingly, triple CNA was validated as a universal prognostic factor responsible for the survival difference between KNBTG and TCGA.

## Discussion

In this study on *IDH*-wild-type GBM, we conducted comparative profiling for CNA status and clinical outcome targeting two large cohorts: KNBTG and TCGA. Firstly, CNA frequencies of *EGFR*, *PTEN*, and *CDKN2A* turned out to exhibit extreme variation between these cohorts. Secondly, the triple overlap in these three loci (triple CNA) proved to be approximately three-fold higher in frequency in TCGA (70.5%) than KNBTG (24.1%). The prognostic impact of this finding was validated in both cohorts, which consisted entirely of GBM patients homogenously treated by standard chemoradiation. Although KNBTG cohort significantly showed the better prognosis than TCGA, its survival difference completely resolved after subclassifying all cases by triple CNA status, in contrast to other common prognostic molecular markers. Consequently, the discrepancies in the distribution of triple CNA in two cohorts could provide a full account of the cohort disparities of survival duration.

The prognostic impact of CNAs is still controversial; however, some of them have the potential to be related with clinical outcomes in GBM. The impact of *NFKBIA* deletion on survival was observed in both KNBTG and TCGA cohorts in the present study, and this result was in line with a previous report [2]. Even though the CNAs in *EGFR*, *CDKN2A*, and *PTEN* affected patients’ clinical outcome insufficiently by itself, these markers proved to commonly show a prognostic impact when combined. In another attempt to provide prognostic assessment based on CNA molecular subtype, the classification defined by chromosome 1 gain, 19 gain, and *CDK4*/*MDM2* co-amplification was recently proposed through an analysis on the dataset from TCGA and German Glioma Network [9]. The prognostic discrimination according to this classification was incompletely validated in *IDH*-wild-type GBM; however, their findings supported the impact of CNAs on survival. These clinical impacts of CNA profiles immediately raise a question of whether any molecular marker could make “authentic” GBMs further distinguishable from preexisting entities diagnosed by histopathological findings and *IDH1/2* status. Our findings support the need to introduce molecular markers in the diagnostic criteria of GBM and re-evaluate the definition of GBMs.

The underlying reason for the unequal distribution of CNAs between Japan and TCGA cohorts is poorly understood; however, there are two presumptive causes. The first hypothesis is the interobserver variances in the histopathological diagnosis of GBMs. In practice, the diagnosis of GBM in our cohort was solely based on the universal guidelines in WHO classification. However, even the universal criteria may pose some limitations, and, hence, molecular marker seems to be of practical value in GBM diagnostics. The second hypothesis is the geographical variances in the frequencies of CNAs. The regional difference has been widely recognized in other malignant tumors such as lung adenocarcinoma [20, 30], breast cancer [14], and endometrial cancer [12]. A well-known example is the high prevalence of *EGFR* mutations in pulmonary adenocarcinoma in Asia. Despite few data concerning GBM, the low prevalence of *TP53* mutations in whites and homozygous deletion of *CDKN2* gene in Japanese were reported [7, 21], along with the regional disproportion of *EGFR* amplification in GBM. These findings are roughly compatible with our results in that the most striking disproportion of genetic aberrations is mainly in the loci of *EGFR*, *CDKN2A*, *PTEN*, and *TP53*. Our study may provide an additional evidence for the interregional molecular disproportion of GBM. Further study is needed to elucidate regional variances and its clinical value.

This is an illustrative cohort study showing that different clinical outcomes across cohorts could be attributed to the uneven distributions of prognostic genetic backgrounds. We formed two independent cohorts consisting of population subjected to the same treatment, and successfully validated the common prognostic value of triple CNA. Notably, the survival discrepancy between the cohorts resolved after subclassifying these two cohorts by triple CNA. In population-based research, treatment options are submitted to physicians in local institutions and depend on the socioeconomical status of patients. Therefore, population-based cohort is reflective of the general population, but is likely to be a heterogeneously treated population, which makes it difficult to elucidate the clinical value of molecular markers. Our results indicate that some underlying selection biases remain even after adjusting the treatment backgrounds, and that one of those biases is the differences in prognostic CNA profiles. Along with our results, Cimino et al. showed an inherent bias that enrolled patients for the prospective cohort in clinical trial are liable to bear favorable molecular markers for survival, using molecular profiles by their original CNA subclassification [8]: the prospective cohort in clinical trials thus, less likely mirrors the general population. Therefore, the potential biases in genetic background including CNA profiles should be taken into consideration while interpreting clinical trials or biomarker studies.

This study harbors the limitations as a retrospective analysis susceptible to selection biases; the initial inclusion criteria for molecular data availability probably reflected one of the selection biases. Instead, the cohorts in this study could be regarded as reflective of the general population with homogeneous treatment background. The other limitation is the different molecular biological techniques utilized in the two cohorts. We evaluated CNAs by MLPA, which is a reliable and cost-effective method, but lower throughput than the comprehensive high-throughput array of the TCGA cohort. The resultant CNA profiling in our cohort seems dependable, because interactive CNA relationships such as co-occurrence and mutual exclusivity showed mostly equivalent outcomes to previous reports (Additional file 1: Figure S1). It is, however, difficult to discriminate by MLPA technique whether the CNA involves entire chromosomal arm or focal locus; low-copy *EGFR* amplification and 7p gain is hard to be distinguished. Nonetheless, the putative copy number calls downloaded from cBioPortal was virtually equivalent to that generated by MLPA because focal gene copy number was equally estimated rather than the entire chromosomal aberrations. Our analyses incorporated hemizygous and homozygous deletion of *CDKN2A* as one of the molecular markers. The biological significance of hemizygous deletion is not relevant in the molecular pathway while *CDKN2A* homozygous deletion is regarded as one of the representative causes for disruptions of Rb1 pathway. When incorporating only homozygous deletion of *CDKN2A* for the definition of triple CNA, the prognostic impact of this combination is retained in the Japan cohort but not in the TCGA cohort (data not shown). Further investigation using homogeneous molecular techniques in independent larger cohorts is needed to validate our findings and select the optimal combination of CNAs. However, we believe that this study adequately addressed the clinical importance and geographical distribution of CNAs in GBMs.

## Conclusions

We conclude that specific CNA profiles harbor significant impacts on the survival of GBM patients, and that the distribution of CNAs potentially affects clinical outcomes across cohorts. We believe that the GBM classification according to copy number profiles would be a prognostic indicator and provide new insight into the interpretation and comparison of interregional clinical trials.

## Additional files

Additional file 1: **Figure S1**. Combinations (or pairs) of genetic alterations showing co-occurrence or mutual exclusivity in KNBTG. **Figure S2**. Kaplan-Meier analysis of overall survival in Step1 between KNBTG and TCGA. (PPTX 281 kb)

Additional file 2: **Table S1**. Dataset of patients with glioblastoma from Kansai Molecular Diagnosis Network for CNS tumors (*n* = 234). **Table S2**. Dataset of patients with glioblastoma from The Cancer Genome Atlas (*n* = 577). **Table S3**. Log odds ratio in pair association analysis for genetic aberrations. **Table S4**. *P*-value from two-tailed Fisher’s exact test in pair association analysis for genetic aberrations. **Table S5**. Univariate analysis for each molecular variable using Cox proportional hazards models in Step2. **Table S6**. Sequences of primers used for amplification and Sanger sequencing. **Table S7**. Sequences of primers used for quantitative methylation specific PCR. (ZIP 256 kb)

## Data Availability

The anonymized datasets analyzed in the present study are provided in supplementary information.

## Abbreviations

CNA: copy number alteration
CNS WHO: WHO Classification of Tumors of the Central Nervous System
GBM: Glioblastoma
HR: hazard ratio
KNBTG: Kansai Molecular Diagnosis Network for CNS tumors
KPS: Karnofsky Performance status
LOR: log odds ratio
MGMT: O^6^-methylguanine-DNA-methyltransferase
MLPA: Multiplex Ligation-dependent Probe Amplification
OS: overall survival
qMSP: quantitative methylation specific PCR
RT: radiotherapy
TCGA: The Cancer Genome Atlas
