# Supplementary material for: Distribution differences in prognostic copy number alteration profiles in IDH-wild-type glioblastoma cause survival discrepancies across cohorts
Source: Acta Neuropathol Commun. 2019 Jun 18;7:15. doi: 10.1186/s40478-019-0749-8 (PMC6580599; doi:10.1186/s40478-019-0749-8)
Supplement: Supplementary file 1 — Figure S1. Combinations (or pairs) of genetic alterations showing co-occurrence or mutual exclusivity in KNBTG. Figure S2. Kaplan-Meier analysis of overall survival in Step1 between KNBTG and TCGA. (PPTX 281 kb) [file 40478_2019_749_MOESM1_ESM.pptx]

## Slide 1
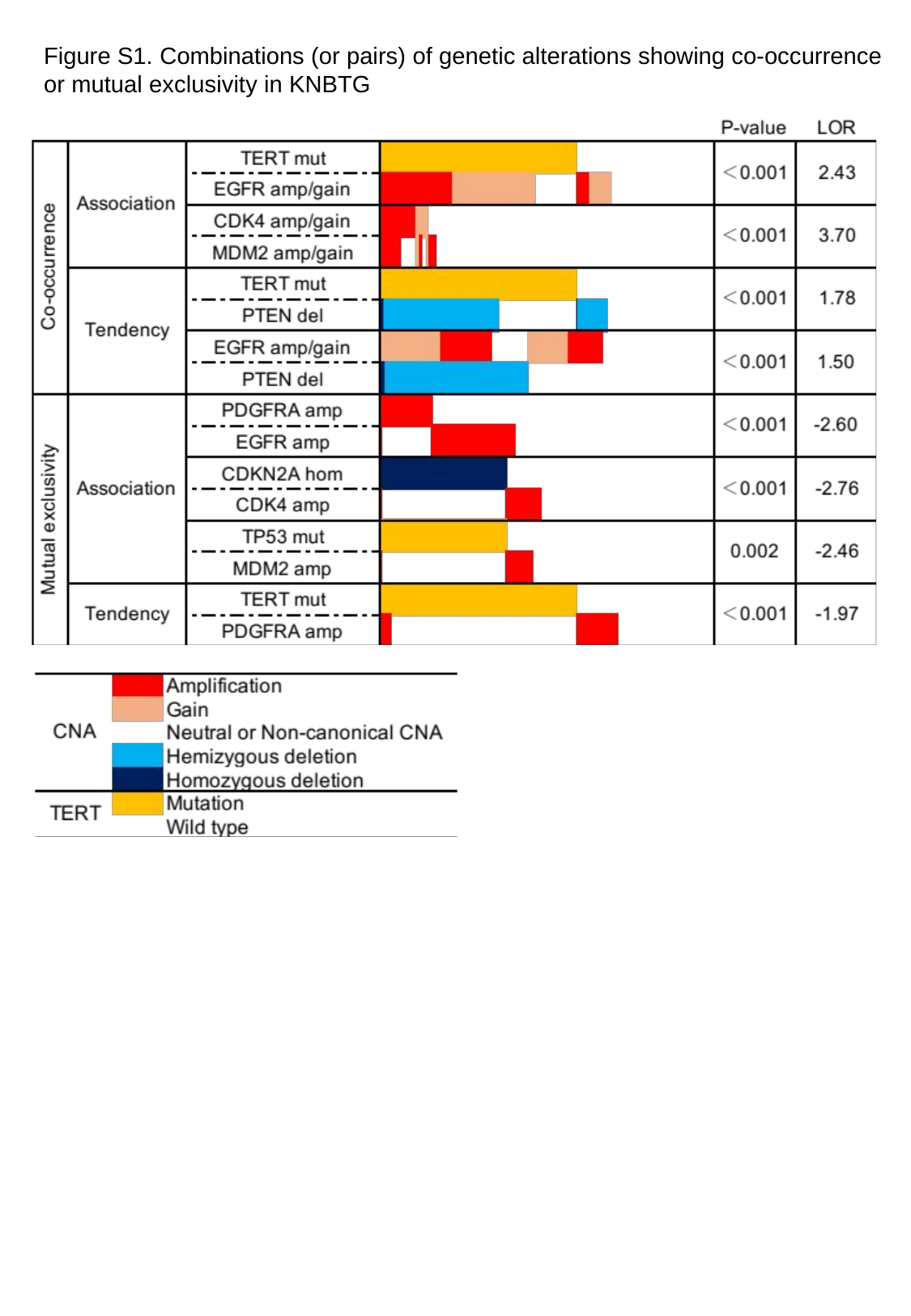

Figure S1. Combinations (or pairs) of genetic alterations showing co-occurrence or mutual exclusivity in KNBTG

## Slide 2
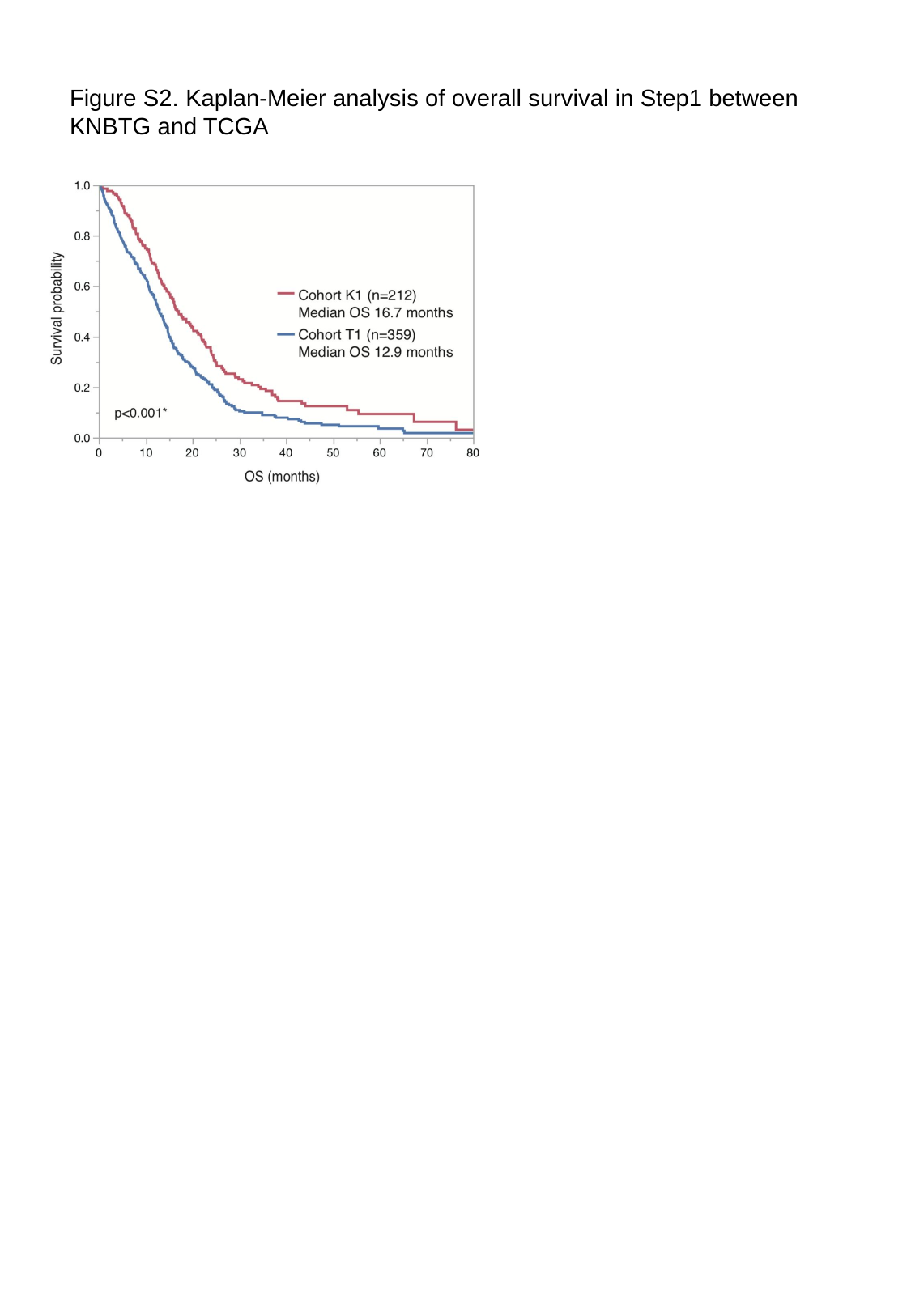

Figure S2. Kaplan-Meier analysis of overall survival in Step1 between KNBTG and TCGA
