# Supplementary material for: Distribution differences in prognostic copy number alteration profiles in IDH-wild-type glioblastoma cause survival discrepancies across cohorts
Source: Acta Neuropathol Commun. 2019 Jun 18;7:15. doi: 10.1186/s40478-019-0749-8 (PMC6580599; doi:10.1186/s40478-019-0749-8)
Supplement: Supplementary file 2 — Table S1. Dataset of patients with glioblastoma from Kansai Molecular Diagnosis Network for CNS tumors (n = 234). Table S2. Dataset of patients with glioblastoma from The Cancer Genome Atlas (n = 577). Table S3. Log odds ratio in pair association analysis for genetic aberrations. Table S4. P-value from two-tailed Fisher’s exact test in pair association analysis for genetic aberrations. Table S5. Univariate analysis for each molecular variable using Cox proportional hazards models in Step2. Table S6. Sequences of primers used for amplification and Sanger sequencing. Table S7. Sequences of primers used for quantitative methylation specific PCR. (ZIP 256 kb) [file 40478_2019_749_MOESM2_ESM.zip › SupplementaryMaterials_ANC_revised.docx]

**Supplementary Information**

**Distribution Differences in Prognostic Copy Number Alteration Profiles in IDH-wild-type Glioblastoma cause survival discrepancies across cohorts**

Authors list

Toru Umehara, Hideyuki Arita, Ema Yoshioka, Tomoko Shofuda, Daisuke Kanematsu, Manabu Kinoshita, Yoshinori Kodama, Masayuki Mano, Naoki Kagawa, Yasunori Fujimoto, Yoshiko Okita, Masahiro Nonaka, Kosuke Nakajo, Takehiro Uda, Naohiro Tsuyuguchi, Junya Fukai, Koji Fujita, Daisuke Sakamoto, Kanji Mori, Haruhiko Kishima, Yonehiro Kanemura

**Supplementary Tables**

### ***Supplementary Table S1. Dataset of patients with glioblastoma from Kansai Molecular Diagnosis Network for CNS tumors (n=234)***

### ***Supplementary Table S2. Dataset of patients with glioblastoma from The Cancer Genome Atlas (n=577)***

### ***Supplementary Table S3. Log odds ratio in pair association analysis for genetic aberrations***

### ***Supplementary Table S4. P-value from two-tailed Fisher’s exact test in pair association analysis for genetic aberrations***

### ***Supplementary Table S5. Univariate analysis for each molecular variable using Cox proportional hazards models in Step2***

### ***Supplementary Table S6. Sequences of primers used for amplification and Sanger sequencing***

### ***Supplementary Table S7. Sequences of primers used for quantitative methylation specific PCR***

**Supplementary Methods**

***Outline of Molecular analysis***

We screened anonymous fresh specimens of 234 primary GBM collected from seven institutions participating in Kansai Molecular Diagnosis Network for CNS Tumors: Departments of Neurosurgery, Osaka University Graduate School of Medicine; Department of Neurosurgery, Osaka International Cancer Institute; Department of Neurosurgery, Osaka National Hospital (ONH), National Hospital Organization; Department of Neurosurgery, Osaka City University Graduate School of Medicine; Department of Neurosurgery, Wakayama Medical University; Department of Neurosurgery, Hyogo College of Medicine; Department of Neurosurgery, Kansai Rosai Hospital. All specimens were resected between December 2006 and November 2017: i.e., since the beginning of temozolomide era in Japan. Tumor genomic DNA was extracted in two laboratories: ONH and Osaka University. All molecular analyses were performed in ONH.

## *Sanger sequence*

The mutations of *IDH1/2*, *TERT* promoter, *H3F3A*, *HIST1H3B*, and *TP53* were assessed by sanger sequencing. Genomic regions of interest were amplified by PCR with gene-specific primers as listed in Supplementary Table S6 [1, 2, 8] and TaKaRa Ex Taq® (Takara Bio Inc., Shiga, Japan) using an Applied Biosystems GeneAmp PCR System 9700 (Thermo Fisher Scientific). Purified PCR products were sequenced with sequencing primer (SEQ-primer) or one of the PCR primers (PCR-primer) and a BigDye® Terminator V1.1 Cycle Sequencing Kit (Thermo Fisher Scientific) using an ABI 3130xL Genetic Analyzer (Thermo Fisher Scientific). Sequences of primers used for amplification or Sanger sequencing are provided in Supplementary Table S6.

***MGMT promoter methylation***

The methylation status of the *MGMT* promoter was assessed using quantitative methylation specific PCR (qMSP). Purified DNA was subjected to bisulfite modification by an EZ DNA Methylation-Gold Kit (Zymo Research, Irvine, CA), according to the manufacturer’s instructions. The qMSP was performed on a QuantStudio12K Flex Real-Time PCR System (Thermo Fisher Scientific) with POWER SYBR® Green PCR Master Mix (Thermo Fisher Scientific). The bisulfite-modified DNA was amplified using specific primer for each methylated or unmethylated molecule as listed in Supplementary Table S7. Real-time PCR conditions were 95 °C for 10 min followed by 45 cycles of 95 °C for 10 s, 60 °C for 60 s. The quantification of methylated and unmethylated sequences was performed by employing the standard curve method as previously described. In dissociation curve analysis, heterogeneity of the amplified methylated and unmethylated molecules was assessed from melting temperature. The mean ± standard deviation of methylation value was calculated from triplicate PCRs. We used 1% cut-off value for the determination of *MGMT* methylation based on an outcome-based study of newly diagnosed GBMs as mentioned in our previous publications[6, 7]. Sequences of primers used for quantitative methylation specific PCR are provided in Supplementary Table S7.

***Multiplex Ligation-dependent Probe Amplification***

　Copy number alterations (CNA) was assessed by Multiplex Ligation-dependent Probe Amplification (MLPA) using the SALSA MLPA KIT P105 (version D2) and P088 (version C2), in accordance with the manufacturer’s protocol (MRC Holland, Amsterdam, Netherland)[4]. Amplification products were separated on an ABI-3130XL Genetic Analyzer (Applied Biosystems, Foster City, CA) and quantified using GeneMapper 5.0 software (Applied Biosystems). Quantitative data at each target region were analyzed using the Coffalyser software, version 140721.1958 (MRC Holland; www.coffalyser.net), where relative probe values of probe-amplified products are compared with normal controls. Abnormal/normal probe value limits were set at 0.7 and 1.3 according to the manufacturer’s recommendations. Based on previous publications, the CNA category was classified by the following thresholds: homozygous deletion (x ≤ 0.4), hemizygous deletion (0.4 < x ≤ 0.7), gain (1.3 ≤ x < 2.0), amplification (x ≥ 2.0)[5]. When probe values within one locus heterogeneously distributed across some categories such as hemizygous and homozygous deletions, we defined the category in which more than 70% of probe values belonged. For convenience, homozygous and/or hemizygous deletion collectively were referred to as deletion, while amplification and/or gain as amp/gain. Non-canonical CNAs such as amp/gain in *CDKN2A*, *PTEN*, *TP53*, or *NFKBIA* and deletion in *PDGFRA*, *EGFR*, *CDK4*, or *MDM2* were not considered in the data analysis.

## *Supplementary Table S6. Sequences of primers used for amplification and Sanger sequencing.*

| gene | primer | sequence |
| --- | --- | --- |
| *IDH1*[2] | PCR-forward | 5′-AATGAGCTCTATATGCCATCACTG-3′ |
|  | PCR-reverse | 5′-TTCATACCTTGCTTAATGGGTGT-3′ |
|  | SEQ-forward | 5′-GCCATCACTGCAGTTGTAGGTTA-3′ |
| *IDH2*[8] | PCR-forward | 5′-TTGTTGCTTGGGGTTCAAAT-3′ |
|  | PCR-reverse | 5′-TGTGGCCTTGTACTGCAGAG-3′ |
| *TERT* promoter[1] | PCR-forward | 5′-TCCCTCGGGTTACCCCACAG-3′ |
|  | PCR-reverse | 5′-AAAGGAAGGGGAGGGGCTG-3′ |
| *H3F3A* (for K27 and G34) | PCR-forward | 5′-GATTTTGGGTAGACGTAATCTTCA-3′ |
|  | PCR-reverse | 5′-TACATACAAGAGAGACTTTGTCCC-3′ |
| *TP53* exon 2-4 | PCR-forward | 5′-CAGGAGTGCTTGGGTTGTGG-3′ |
|  | PCR-reverse | 5′-AGAAATGCAGGGGGATACGGC-3′ |
| *TP53* exon 5-9 | PCR-forward | 5′-TGCCCTGACTTTCAACTCTG-3′ |
|  | PCR-reverse | 5′-GTTAGCTACAACCAGGAGCCA-3′ |
| *TP53* exon 10-11 | PCR-forward | 5′-ATGCATGTTGCTTTTGTACCG-3′ |
|  | PCR-reverse | 5′-TGTTCTGACGCACACCTAT-3′ |
| *TP53* exon 6 | SEQ-reverse | 5′-CTACTGCTCACCCGGAGG-3′ |
| *TP53* exon 7 | SEQ-forward | 5′-CCACAGGTCTCCCCAAGG-3′ |

## *Supplementary Table S7. Sequences of primers used for quantitative methylation specific PCR.*

| gene | primer | sequence |
| --- | --- | --- |
| *MGMT* promoter[3] | M-forward | 5′-TTTCGACGTTCGTAGGTTTTCGC-3′ |
|  | M-reverse | 5′-GCACTCTTCCGAAAACGAAACG-3′ |
|  | U-forward | 5′-TTTGTGTTTTGATGTTTGTAGGTTTTTGT-3′ |
|  | U-reverse | 5′-AACTCCACACTCTTCCAAAAACAAAACA-3′ |
| M, Methylated; U, Unmethylated | | |

# Supplementary figures

## *Supplementary Figure S1. Sets of genetic alterations with co-occurrence or mutual exclusivity*

***Supplementary Figure S2. Kaplan-Meier analysis of overall survival in Step1 between KNBTG and TCGA***

# Supplementary Figure Legends

## *Supplementary Figure S1. Combinations (or pairs) of genetic alterations showing co-occurrence or mutual exclusivity in KNBTG*

The heatmap shows sets of genetic alterations bearing association and tendency toward co-occurring or mutual exclusivity. Concurrent with significant correlation (p < 0.05, Fisher’s exact test), the log odds ratio (LOR) below -2.0 and above 2.0 was respectively defined as association toward mutual exclusivity and co-occurrence. LOR from -2.0 to -1.5 and 1.5 to 2.0 was independently defined to have a tendency of these associations. Association toward co-occurrence was observed between *TERT* promoter mut and *EGFR* amp/gain (LOR 2.43, p < 0.001). The pair of *MDM2* and *CDK4*also had an association toward co-amplification (LOR 3.70, p < 0.001).

*PTEN* del has a tendency of co-occurrence with *TERT* promoter mut (LOR 1.78, p < 0.001) and *EGFR* amp/gain (LOR 1.50, p < 0.001). Association toward mutual exclusivity was observed in the sets of *PDGFRA* amp and *EGFR* amp (LOR -2.60, p < 0.001), *CDKN2A* del and *CDK*4 amp (LOR -2.76, p < 0.001), and *TP53* mut and *MDM2* amp (LOR -2.46, p = 0.002). *TERT* promoter mut has a tendency of mutual exclusivity with *PDGFRA* amp (LOR -1.97, p < 0.001).

Abbreviations: amp, amplification; hom, homozygous deletion; mut, mutation

## *Supplementary Figure S2. Kaplan-Meier analysis of overall survival in Step1 between KNBTG and TCGA*

Kaplan–Meier analyses of OS in Step1 for Cohort K1 (n = 212) and T1(n = 359) are shown. OS of Cohort T1 was significantly shorter than that of Cohort K1 (p < 0.001, Log-rank test), albeit disregarding the treatment background.

# References

1 Arita H, Narita Y, Fukushima S, Tateishi K, Matsushita Y, Yoshida A et al (2013) Upregulating mutations in the TERT promoter commonly occur in adult malignant gliomas and are strongly associated with total 1p19q loss. Acta Neuropathol 126: 267-276 Doi 10.1007/s00401-013-1141-6

2 Bleeker FE, Lamba S, Leenstra S, Troost D, Hulsebos T, Vandertop WP et al (2009) IDH1 mutations at residue p.R132 (IDH1(R132)) occur frequently in high-grade gliomas but not in other solid tumors. Human mutation 30: 7-11 Doi 10.1002/humu.20937

3 Esteller M, Hamilton SR, Burger PC, Baylin SB, Herman JG (1999) Inactivation of the DNA repair gene O6-methylguanine-DNA methyltransferase by promoter hypermethylation is a common event in primary human neoplasia. Cancer Res 59: 793-797

4 Jeuken J, Cornelissen S, Boots-Sprenger S, Gijsen S, Wesseling P (2006) Multiplex ligation-dependent probe amplification: a diagnostic tool for simultaneous identification of different genetic markers in glial tumors. J Mol Diagn 8: 433-443 Doi 10.2353/jmoldx.2006.060012

5 Jeuken J, Sijben A, Alenda C, Rijntjes J, Dekkers M, Boots-Sprenger S et al (2009) Robust detection of EGFR copy number changes and EGFR variant III: technical aspects and relevance for glioma diagnostics. Brain Pathol 19: 661-671 Doi 10.1111/j.1750-3639.2009.00320.x

6 Okita Y, Nonaka M, Shofuda T, Kanematsu D, Yoshioka E, Kodama Y et al (2014) (11)C-methinine uptake correlates with MGMT promoter methylation in nonenhancing gliomas. Clinical neurology and neurosurgery 125: 212-216 Doi 10.1016/j.clineuro.2014.08.004

7 Sasaki T, Fukai J, Kodama Y, Hirose T, Okita Y, Moriuchi S et al (2018) Characteristics and outcomes of elderly patients with diffuse gliomas: a multi-institutional cohort study by Kansai Molecular Diagnosis Network for CNS Tumors. J Neurooncol: Doi 10.1007/s11060-018-2957-7

8 Yan H, Parsons DW, Jin G, McLendon R, Rasheed BA, Yuan W et al (2009) IDH1 and IDH2 mutations in gliomas. N Engl J Med 360: 765-773 Doi 10.1056/NEJMoa0808710
